# Miracle Tree *Moringa oleifera*: Status of the Genetic Diversity, Breeding, In Vitro Propagation, and a Cogent Source of Commercial Functional Food and Non-Food Products

**DOI:** 10.3390/plants11223132

**Published:** 2022-11-16

**Authors:** Hemasundar Alavilli, Yugandhar Poli, Kumar Sambhav Verma, Vikram Kumar, Swati Gupta, Vigi Chaudhary, Anupam Jyoti, Shivendra V. Sahi, Shanker Lal Kothari, Ajay Jain

**Affiliations:** 1 Department of Bioresources Engineering, Sejong University, Seoul 05006, Republic of Korea; 2 ICAR-Indian Institute of Rice Research, Hyderabad 500030, India; 3 Amity Institute of Biotechnology, Amity University Rajasthan, Jaipur 303002, India; 4 Department of Biosciences, Manipal University Jaipur, Jaipur 303007, India; 5 Biotechnology Department, Chandigarh University, National Highway-95, Ludhiana-Chandigarh State Highway, Chandigarh 160055, India; 6 Department of Biology, Saint Joseph’s University (University City Campus), 600 South 43rd Street, Philadelphia, PA 19104, USA

**Keywords:** *Moringa oleifera*, germplasm, genetic diversity, breeding, in vitro propagation, functional food and non-food products, trade, health concerns

## Abstract

*Moringa oleifera* Lam. (MO) is a fast-growing drought-resistant tree belonging to the family Moringaceae and native to the Indian subcontinent and cultivated and/or naturalized worldwide with a semi-arid climate. MO is also popularly known as a miracle tree for its repertoire of nutraceutical, pharmacological, and phytochemical properties. The MO germplasm is collected, conserved, and maintained by various institutions across the globe. Various morphological, biochemical, and molecular markers are used for determining the genetic diversity in MO accessions. A higher yield of leaves and pods is often desirable for making various products with commercial viability and amenable for trade in the international market. Therefore, breeding elite varieties adapted to local agroclimatic conditions and in vitro propagation are viable and sustainable approaches. Here, we provide a comprehensive overview of MO germplasm conservation and various markers that are employed for assessing the genetic diversity among them. Further, breeding and in vitro propagation of MO for various desirable agronomic traits are discussed. Finally, trade and commerce of various functional and biofortified foods and non-food products are enumerated albeit with a need for a rigorous and stringent toxicity evaluation.

## 1. Introduction

**Figure 1:**
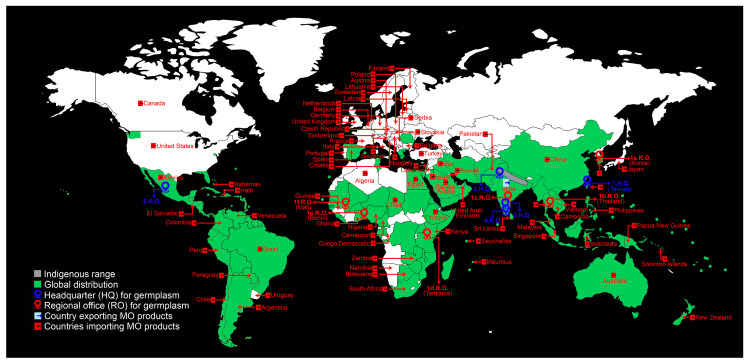
Global distribution of MO (native and/or naturalized), centers for germplasm conservation, and countries importing Moringa products from India.

The monogeneric family Moringaceae comprises phenotypically varied 13 species including *Moringa oleifera* Lam. (MO), *M. concanensis* (MC), and *M. peregrine* (MP) [1]. The phylogenetic analysis based on the geographic ranges and different traits (morphological, anatomical, biochemical, and molecular) revealed the proximity of MO and MC and their divergence from the common ancestor MP [1,2]. MO is commonly known as ben oil tree, drumstick tree, or horseradish tree and is indigenous to northwest India and is now cultivated and/or naturalized in semi-arid, tropical, and subtropical regions of several countries in Africa, Asia, Australia/Oceania, Europe, North America, Central America, and South America, and the Caribbean (Figure 1). MO’s wide assortment of culinary, nutraceutical, pharmacological, and phytochemical properties, providing remedies for various ailments and chronic diseases, and applications in biofuel production, environmental management, and water treatment has earned it several epithets (crops for the future, natural gift, mother’s best friend, and never die) [3,4,5,6,7,8,9,10]. MO plays an important role in the United Nations Sustainable Development Goal 2 (SDG 2) to achieve food security by shifting towards improved nutrition and healthier diets from the varied sustainable agricultural system [11]. Further, the African Orphan Crops Consortium (AOCC) was established to work on 101 chosen underutilized crops of Africa, including MO, to alleviate the ever-growing problem of hidden hunger (food deficient in minerals and vitamins) and encourage the resilient and nutritious agri-food system for the acutely undernourished African population [12].

MO is a fast-growing (5–12 m tall), drought-tolerant, deciduous-to-evergreen perennial, and raised from seeds or cuttings in different agroclimatic regions. MO has a whitish-grey fissured and glabrous bark with a thick cork exuding reddish-brown thermostable gum with potential pharmaceutical properties [13,14]. The density of the soft and light wood is ~0.5–0.7 g/cm^3^ (www.worldagroforestry.org; accessed on 15 February 2022) and its specific gravity (0.28 ± 0.03) was reported to be the lowest among 71 tree species grown in the tropical forests at Kolli hills in central Tamil Nadu, India [15]. The wood is largely used as fuel and sporadically for construction works. Tripinnate (30–60 cm long) pale green leaves comprising several small leaflets (1.3–2.0 cm long and 0.3–0.6 cm wide) are arranged spirally and alternate on pubescent rachis (12–25 cm long) and provide feathery foliage with a pungent odor of a horseradish. The bisexual zygomorphic flower comprises stamens (five fully formed fertile alternating with 5–7 smaller sterile), gynoecium (unilocular and tricarpellary with a slightly curved hollow style, and a minute stigma), corolla (five yellowish-white petals and one of them is erect to which the anthers are joined and four are deflexed), and calyx (deeply five-partite and liner-lanceolate, and refluxed sepals). The fragrant flowers are axillary and form 10–25 cm long scattering panicles and blooms throughout the year. The pods are ~20–50 cm long and pendulous containing papery-winged seeds dispersed by water and wind over a short distance. In MO, the pistil grows beyond the anthers a few hours after anthesis, and delayed stigma receptivity favor cross-pollination mediated by bees, insects, and sunbirds, and exhibits both geitonogamy and xenogamy. Pollen is dispersed within 20 m, which suggests an effective pollen-mediated gene flow to a rather limited distance [16]. MO is conventionally propagated either by limb cuttings or by seeds, and its commercial production in different agro-climatic ecosystems is warranted for alleviating undernourishment in developing countries and augmenting food security [17,18,19]. However, MO is prone to various biotic and abiotic stresses [20]. Harnessing the diversity in natural genetic resources for breeding programs is an attractive paradigm for circumventing multiple biotic and abiotic stresses in plant species [21]. Although plant propagates easily by seeds and stem cuttings, the percent of seed germination is often low, and the plants grown from the stem cuttings show high mortality and low yield [13,14,22]. Moreover, the susceptibility of MO to several insect infestations and fungal infections leads to foliar damage and loss of biomass exerting an adverse influence on the overall yield and nutritional values [23]. Therefore, in vitro propagation of MO provides a viable alternative for both conservation and clonal multiplication of genetically identical and infection-free elite germplasm year-round in a limited space and time under controlled growth conditions to produce plant tissues to meet the ever-growing global demand [13,14,22].

Bibliometrics employs mathematical and statistical tools for the quantitative and qualitative analysis of publications [24]. In 2004, Elsevier launched Scopus (www.scopus.com; accessed on 15 February 2022), which is an abstract and citation database that provides a quality measure in terms of cite score and has broad journal coverage, especially in science, technology, and medicine. Scopus database is now routinely used for the bibliometric analysis of the research and review publications in diverse areas. Therefore, the Scopus database was used for the bibliometric analysis of the reviews published on genetic diversity in MO in the last 20 years. The search revealed only one review published so far about the cultivation, genetics, ethnopharmacology, phytochemistry, and pharmacology of MO leaves [3]. Since this review, several studies have now been published in the following years on the analysis of the genetic diversity in MO. Therefore, in this review, we provide a comprehensive account of the various morphological, biochemical, phytochemical, and molecular traits employed for assessing the genetic diversity among the accessions of MO collected from different agro-climatic regions across the globe for the identification of elite germplasm. Further, we enumerated breeding and in vitro propagation strategies for developing MO varieties with desirable agronomic traits. Finally, the trade of functional food and non-food products and the need for their stringent toxicity evaluation before their use are discussed. 

## 2. Phylogeny of MO with Other Tree Species with Published Genome Sequences

**Figure 2:**
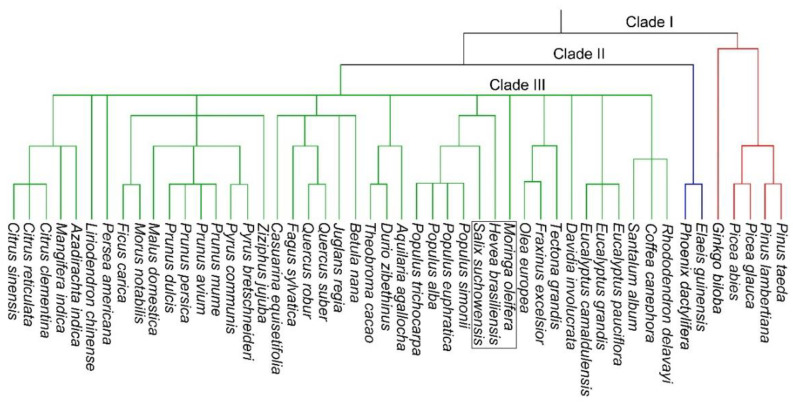
Phylogenetic tree of the 50 tree species with published genome sequences. The topology is based on the total molecular information available at the taxonomy browser of NCBI (https://www.ncbi.nlm.nih.gov/Taxonomy/CommonTree/wwwcmt.cgi; accessed on 20 February 2022).

The draft genome of MO was sequenced on the Illumina platform with 231 MB (~80%) of the total sequence containing more than 19,000 protein-coding genes and was a significant achievement for its improvement by biotechnological interventions [25,26,27]. In about the last two decades, the reference genome sequences of more than 50 tree species including MO have now been published [28,29]. Reference genome sequences are key to the discovery of functionally diverse genes associated with vegetative and reproductive growth traits, responses to different biotic and abiotic stresses, and ecological adaptations [28,29]. Extensive collinearity and synteny have been observed among tree genomes across different genera [28,29]. With the rapid advances in sequencing technology, tree reference genome sequencing is now proceeding rapidly and many more of them are anticipated to be completed soon [28,29]. A phylogenetic tree of 50 tree species with published genome sequences was constructed by using the molecular information available at the taxonomy browser of NCBI (https://www.ncbi.nlm.nih.gov/Taxonomy/CommonTree/wwwcmt.cgi; accessed on 20 February 2022) (Figure 2). The analysis revealed the clustering of 50 tree species into three distinct clades and phylogenetic closeness of MO with *Hevea brasiliensis* and *Olea europea* belonging to the family Euphorbiaceae and Oleaceae, respectively (indicated in a box). 

## 3. Abiotic and Biotic Stresses

### 3.1. Abiotic

The drought-tolerant MO can adapt and grows well in areas with a wide range of altitudes (below 600 m up to 1200 m in the tropics), annual rainfall (~250–1500 mm), and temperatures (25–35 °C) but can also tolerate light frost and higher temperature (~48 °C) in the shade. It prefers a well-drained sandy loam to clay loam but is susceptible to waterlogged soil and poor drainage. Abiotic stresses induced antioxidant metabolites in MO callus [30].

### 3.2. Biotic

Although MO is generally resistant to most pests, many of them have been reported to infect during its growth under certain agro-climatic conditions across the globe [20]. MO also suffers from fruit rot, twig canker, and powdery mildew caused by fungal pathogens *Cochliobolus hawaiiensis*, *Fusarium pallidoroseum*, and *Leveillula taurica,* respectively [20]. Several pathogenic fungi (*A. flavus*, *A. niger*, *Alternaria alternate*, *Fusarium oxysporum*, *Macrophomina phaseolina*, and *Rhizopus stolonifera*) have also been reported from the harvested pods, which are sold on the market [20]. An endophytic fungus colonizes the tissues of its host plant without showing any apparent symptoms of a disease. Several fungal endophytes (*Aspergillus*, *Fusarium*, *Macrophomina*, *Nigrospora*, *Pestalotiopsis*, *Rhizoctonia*, *Stachybotrys*, and *Xylaria*) were also found to be associated with the leaves of MO [31]. MO is also susceptible to various pests (aphids (*Aphis gossypii*), bark borer (*Indarbela tetraonis*), bark-eating caterpillar (*Indarbela quadrinotata* and *I. tetraonis*), beetle (*Batocera rubus *and Myllocerus** spp.), black hairy caterpillar (*Pericallia ricini*), budworm (*Noorda moringae*), bug midge (*Stictodiplosis moringae*), grasshopper (*Homorocoryphus nitidulus*, *Oedaleus nigeriensis*, and *Zonocerus variegates*), green leaf caterpillar (*Noorda blitealis*), hairy caterpillar (*Eupterote mollifera*, *Metanastria hyrtaca*, *Pericallia ricini,* and *Streblote siva*), house fly (*Musa domestica*), insects (*Ceroplastodes cajani and Ulopeza phaeothoracica)*, leaf caterpillar (*Lepidoptera* sp., *Noorda blitealis*, and *Pyralidae* sp.), leaf-eating beetle (*Lagria villosa*), leaf-eating weevils (*Myllocerus* spp.), leaf-footed bug (*Leptoglossus phyllopus*), moth (*Eupterote mollifera and Noorda blitealis*), longhorn beetle (*Batocera rubus*), pod fly (*Gitona distigma*), red spider mite (*Tetranychus urticae*), red wood ant (*Formica rufa*), termites, and whiteflies (*Bemisia* spp.) causing minor or occasionally serious damage [20]. 

## 4. Germplasm

There has been a global effort to collect, conserve, and exchange MO germplasm, which is pivotal for identifying elite accessions that could be used for multiplication in different agroclimatic regions and/or for breeding programs for developing desirable horticultural traits. The World Vegetable Center (WorldVeg), earlier known as the Asian Vegetable Research and Development Center (AVDRC) (https://avrdc.org; accessed on 1 March 2022), is an autonomous international non-profit agricultural research center with headquarters at Shanhua in Taiwan and several regional offices in East Asia (National Institute of Horticultural and Herbal Science (NIHHS) at Jeonbuk in Korea), Southeast Asia (Kasetsart University’s Kamphaeng Saen Campus at Nakhon Pathom in Thailand), South Asia (International Crops Research Institute for the Semi-Arid Tropics (ICRISAT) Campus at Patancheru in India), East Africa (at Arusha in Tanzania), and West Africa (Samanko Research Station at Bamako in Mali and International Institute of Tropical Agriculture (IITA) at Cotonou in Benin) (Figure 1) (https://avrdc.org; accessed on 1 March 2022). The information on MO could also be retrieved from the Global Biodiversity Information Facility (GBIF), which is an international organization providing scientific data on biodiversity using web services (https://www.gbif.org/species/3054181; accessed on 1 March 2022). The WorldVeg maintains the germplasm, mostly in the form of seeds for the improvement and development of new vegetable cultivars (https://avrdc.org/seed/unimproved-germplasm; accessed on 1 March 2022). In 2001, WorldVeg initiated research on Moringa and possesses a collection of over 50 accessions, the majority of which belong to MO collected from Africa, Asia, and the USA, and has distributed the germplasm to several countries in Africa (Egypt), Asia (Malaysia, Pakistan, Philippines, Thailand, and Vietnam), and Europe (Germany and Netherlands) [32]. Several accessions of MO collected from India, Laos, Philippines, Taiwan, Tanzania, Thailand, and the USA and maintained at WorldVeg were also evaluated in the Philippines and Taiwan for promising horticultural traits with the potential of improving the nutrition qualities [33]. From the foothills of the western Himalayas, 23 accessions of MO including one cultivated type were collected and conserved at the Indian Council of Agricultural Research-National Bureau of Plant Genetic Resources (ICAR-NBPGR), New Delhi (Figure 1) (http://www.nbpgr.ernet.in; accessed on 1 March 2022). ICAR-NBPGR performed extensive exploration and germplasm collection of selected vegetable crops including MO (257 accessions) from 1976 to 2018 [34]. Details of nine accessions of MO are also available through the ICAR-NBPGR Cryo Database (http://www.nbpgr.ernet.in:8080/cryobank; accessed on 1 March 2022). In India, a rich repository of MO accessions (~70–90) is also being maintained at Tamil Nadu Agricultural University (TNAU) at Periyakulam, (Figure 1) (https://agritech.tnau.ac.in; accessed on 1 March 2022) and The Indian Council of Agricultural Research-Indian Institute of Horticultural Research (ICAR-IIHR) at Bengaluru (Figure 1) (https://www.iihr.res.in; accessed on 1 March 2022). In China, a gene bank containing 356 germplasm covering 13 *Moringa* species has been established [35]. The International *Moringa* Germplasm Collection at the Instituto de Biología, Universidad Nacional Autónoma de México (UNAM), Jalisco, Mexico maintains the germplasm of MO for both basic and applied scientific research (Figure 1) (https://www.moringaceae.org/index.html; accessed on 1 March 2022).

## 5. Assessment of Genetic Diversity

The genetic diversity of plant species is the fundamental source for variations in the traits, which increases adaptability and consequently expands their geographical distribution [36,37]. Cross-pollinated and entomophilous MO has been widely introduced and naturalized in tropical and sub-tropical countries across the globe and is thus anticipated to have a broad genetic diversity [38]. The extent of genetic diversity among the individuals of a species could be quantified by assaying an array of morphological, biochemical, and molecular traits [36,39].

### 5.1. Morphological Traits

Conventionally, various vegetative and reproductive morphological traits have been used to identify the taxa, evaluate the systematic position, and differentiate cultivars or accessions [36]. A descriptors list of the selected morphological characters (habit, bark color, leaves, leaflets shape, receptacle, flower color, flower symmetry, sepals, petals, anthers, seed, seed cover, and pod length) was recommended for discriminating among the accessions of MO and for generating a character state matrix [40]. In 2007, International Plant Genetic Resources Institute (IPGRI) developed uniform standards for coding, data recording, and scoring for crops. Therefore, based on the guidelines provided by IPGRI, a more comprehensive list of morphological characters (14 qualitative and 11 quantitative) and 48 other descriptors were prepared for the characterization and evaluation of MO accessions [41]. Distinctness, uniformity, and stability (*DUS*) descriptors have also been used for assaying the diversity in MO genotypes [42]. The genetic diversity and population structure of several cultivated and non-cultivated accessions collected from the different *geographical regions across the globe* (Ethiopia, India, Indonesia, Laos, Philippines, Saudi Arabia, Taiwan, Tanzania, Thailand, and the USA) were assayed by using various morphological and *horticultural* traits [32,33,40,41,42,43,44,45,46,47,48,49,50,51,52,53]. These studies revealed the efficacy of the morphological traits in determining the genetic diversity among the accessions and thus facilitated the selection of those with desirable characteristics for the future improvement program of MO. However, the morphological markers are limited in number, and often influenced by the growth and development stages of the plants and various environmental factors [36].

### 5.2. Biochemicals, Nutritional and Anti-Nutritional Elements

Antioxidants (β-carotene and vitamins A, C, and E), biochemicals (amino acids, chlorophyll, glucosinolates, seed protein, sugars, and total protein), heavy metals, in vitro gas production, macronutrients (calcium [Ca], magnesium [Mg], nitrogen [N], phosphorus [P], and potassium [K]), micronutrients (copper [Cu], iron [Fe], manganese [Mn], and zinc [Zn]), nutritional and anti-nutritional factors (lead [Pb], oligosaccharides, and oxalate), and polyphenols (baicalin, caffeic acid, chlorogenic acid, cinnamic acid, coumaric acid, ferulic acid, gallic acid, gallogen, isoquercetin, kaempferide, quercetin, quercitrin, rutin, and vanillin) have been employed for determining the genetic variability among the accessions and/or advanced breeding lines of MO from China, India, Laos, Philippines, Saudi Arabia, Taiwan, Tanzania, Thailand, and the USA [32,54,55,56,57,58,59] (Table 1). However, like morphological markers, biochemical markers also have several limitations, i.e., their fewer numbers, low efficacy in detecting polymorphism and being affected by the growth and developmental stages of the plant, and various biotic and abiotic stresses [36]. 

### 5.3. Molecular Traits

Molecular markers provide an attractive paradigm as they are independent of environmental fluctuations, conducive to automation, wider genomic coverage, and high reproducibility [36,60]. Depending on the method of analysis, the molecular markers are categorized into hybridization-based (e.g., restriction fragment length polymorphism (RFLP)), polymerase chain reaction (PCR)-based (e.g., random amplified polymorphic DNA (RAPD)), and sequencing-based (e.g., single nucleotide polymorphisms (SNPs)) [36]. Molecular markers are able (codominant) or unable (dominant) to distinguish the allelic differences of a gene in a heterozygous condition. Although an array of molecular markers is available, each one of them has its specific advantages and limitations. Therefore, several molecular markers were used to assess their efficacy in quantifying the genetic diversity in MO accessions collected from different agroclimatic regions across the world (Table 1).

#### 5.3.1. Amplified Fragment Length Polymorphism (AFLP)

AFLP is a robust and reliable dominant marker for fingerprinting genomic DNAs without prior sequence information by the selective PCR amplification of the restriction fragments of the digested genomic DNA [61]. AFLP was used for analyzing the genetic diversity across 140 accessions of MO collected from India, Kenya, and Malawi, which revealed significant variations between populations and regions [62]. The study suggested that provenance source is crucial for the conservation and exploitation of MO. However, the requirement of high molecular weight DNA and the variation in the precision of the fragment sizes which results in low reproducibility are some of the limitations of the AFLP technique.

#### 5.3.2. Inter-Simple Sequence Repeat (ISSR)

The ISSR technique involves anchoring the designed primers for amplification of the region between oppositely oriented and closely spaced simple sequence repeats (SSRs) [63]. The dominant ISSR marker is potent for marking gene-rich regions [64]. ISSR markers generated a high percentage of polymorphism (~20–91%), average heterozygosity, marker index, and multiplex ratio, and were thus efficacious in revealing extensive genetic diversity and population structure across MO accessions collected from different regions of India [49,65,66], Egypt [67], and Saudi Arabia [48]. One of the limitations of ISSR markers is their low resolution on the agarose gel, which could be significantly enhanced by separating them on a non-denaturing polyacrylamide gel. Since ISSR is a multilocus technique, the non-homology of similar-sized fragments could be disadvantageous.

#### 5.3.3. RAPD

RAPD is a PCR-based technique which does not require sequence information and radioactive probes, employs short (decamer) and random oligonucleotide primers; DNA fragments separated by agarose gel electrophoresis are visualized by staining with ethidium bromide, allowing detection of several loci (0.5 kb to 5 kb) in the genome revealing DNA polymorphism between the individuals [68]. RAPD has become a favored dominant marker due to its simplicity, cost-effectiveness, and efficacy. Therefore, it is not surprising that the versatile RAPD technique was employed in ~50% of the studies involving the use of molecular markers for the detection of genetic diversity (~48–96%) in MOII accessions collected from the diverse geographical regions of Brazil, India, Indonesia, Malaysia, Pakistan, Nigeria, Taiwan, Tanzania, Thailand, and the USA [49,54,55,58,65,69,70,71,72,73,74] (Table 1). However, the presence of false-positive, non-reproducibility, sensitivity to experimental conditions, and the requirement of a high concentration of agarose gel for a better resolution are some of the innate problems with the RAPD technique [36].

#### 5.3.4. Start Codon Targeted (SCoT) Polymorphism

SCoT polymorphism is a novel and simple technique for generating gene-targeted plant DNA markers based on the short, conserved region flanking the translation initiation start codon (ATG) of the plant genes [75]. This technique employs the same single 18-mer primers (forward and reverse) in a PCR reaction at an annealing temperature of 50 °C and the amplicons are separated by agarose gel electrophoresis. Dominant SCoT markers generate high percent polymorphisms with reproducible fingerprint profiles and have been widely used for assessing the genetic diversity, gene flow, and population structure in plant species and for developing co-dominant sequence characterized amplified regions (SCAR) markers [76,77,78]. SCoT markers were used to assess the genetic diversity among the 10 accessions of MO grown naturally in the Middle Delta, Egypt, which revealed ~20% polymorphism [67]. Although SCoT markers are normally reproducible, annealing temperature and primer length are not the only factors governing reproducibility [75].

#### 5.3.5. Internal Transcribed Spacer (ITS)

ITS is the spacer DNA situated between the small and large subunits of ribosomal RNA (rRNA) and is used for assessing genetic diversity and/or phylogenetic relationships [79]. ITS was employed as barcoding marker to assess the genetic diversity among the 10 accessions of MO grown naturally in the Middle Delta, Egypt [67].

#### 5.3.6. SCAR Markers

SCAR marker is a PCR-based technique of cloning and sequencing the termini of selected unique dominant RAPD multilocus markers for designing the flanking 24-mer oligonucleotide forward and reverses primers for the amplification of a single locus [80]. This technique often converts dominant RAPD markers into codominant, locus-specific, highly reliable, and reproducible SCAR markers or polymorphism could also be retained as the presence or absence of the amplified band [80]. The potential efficacy of SCAR markers for the authentication of medicinal herbs used in traditional formulations was critically reviewed [81]. An exploration survey was conducted in the southern states (Karnataka, Kerala, and Tamil Nadu) of India, which led to the identification of 23 accessions (Candidate Plus Trees (CPTs)) with 50% higher fruit yield and single fruit weight than average [52]. RAPD analysis of CPTs revealed a unique band (1.5 kb), which was used for generating stable and specific dominant SCAR makers since each of them amplified only one fragment [52]. SCAR marker was also found to be dominant in asparagus (*Asparagus officinalis* L.) and linked to the sex expression locus *M* facilitating scoring male and female progeny in the mapping population [82]. However, in batay (*Paraseriathes falcataria* [L.] Nielsen), five SCAR markers produced both dominant and codominant polymorphisms [83]. One of the limitations of the SCAR marker technique is the need for sequence data to design the primers. 

#### 5.3.7. Sequence-Related Amplified Polymorphism (SRAP)

SRAP marker technique is a simple and efficient technique which involves the amplification of the open reading frames (ORFs) by using 17–18-mer oligonucleotide comprising core sequences at the 5′ end (13–14-mer oligonucleotide of which first 10–11 are different filler sequences with no specific constitution followed by the sequences CCGG and AATT in the forward and reverse primers, respectively) and three selective nucleotides at the 3′ end [84]. For amplification, the annealing temperature is set at 35 °C for the first five cycles followed by 35 cycles at 50 °C, the amplified DNA fragments are separated by denaturing acrylamide gels and detected by autoradiography [84]. Twenty percent of the SRAP markers were co-dominant in recombinant inbred and doubled-haploid lines of *Brassica oleracea* L. and were also easily amplified in other crop species [84]. Dominant SRAP markers are potent for elucidating the genetic variability in different taxa, constructing linkage maps, and identification of quantitative trait loci (QTL). SRAP markers were used for determining the genetic diversity and population structure of MO accessions of which 97 (95 naturally grown traditional varieties and two hybrids) were collected from Indian states (Andhra Pradesh, Odisha, and Tamil Nadu) [66] and 10 from different islands in the Indonesian archipelago [53]. SRAP markers revealed ~70–81% polymorphism among the accessions from India and Indonesia [53,66].

#### 5.3.8. SSRs

SSRs, also known as microsatellites, are tandem repeats of di- tri- tetra- and pentanucleotide units dispersed ubiquitously throughout the eukaryotic genomes [85]. SSRs are one of the most used molecular markers for studying genetic diversity and population structure due to their codominant inheritance, multiallelic nature, relative abundance, and reproducibility. The genetic diversity of more than 300 accessions of MO collected from the diverse geographical regions across Africa, Asia, the Caribbean, North America, and South America were assayed by SSR technology, which revealed ~80–90% polymorphism [45,47,50,57,72,86,87,88,89]. However, SSR technology is an expensive and time-consuming process and has the limitation of detecting only the sequence repeats.

#### 5.3.9. Cytochrome P450 (CytP450)

CytP450 is a large superfamily of more than 750 heme thiolate proteins responsible for catalyzing monooxygenation of various reactions involved in metabolic pathways and detoxification of xenobiotics in all organisms [90]. Variations in the sequences of CytP450 gene analogs in different plant species offer a useful tool for determining the genetic diversity across the functional regions of the higher plants. Seven pairs of cytochrome P450-based markers generated 40% polymorphism among 8 MO cultivars [65]. Further, ten CytP450 gene-based markers were used for determining the extent of genetic diversity in seven advanced breeding lines of MO developed at different places in the southern state (Karnataka) of India, which revealed ~86% polymorphism and could thus be exploited for the breeding program, cultivar development, DNA fingerprinting, and marker-assisted selection [55]. In another study, seven CytP450 gene-based markers exhibited ~88% polymorphism in 23 accessions collected from the southern states (Karnataka, Kerala, and Tamil Nadu) of India [91]. Together, these studies highlighted the efficacy of CytP450 gene-based markers in elucidating the genetic diversity in MO accessions [55,65,91]. 

**Table 1:** Morphological, biochemical, and molecular markers used for assaying the genetic diversity in MO accessions.

S. No.	Source of genotypes	Number of accessions assayed	Morphological	Biochemical and phytochemical	Molecular									References
					AFLP	ISSR	RAPD	SCoT	ITS	SCAR	SRAP	SSR	CytP450	
1	Cuba, India, Kenya, Mali, Myanmar, Taiwan, and USA.	11												[89]
2	India	32												[42]
3	India	23												[51]
4	India	55												[50]
5	India	23												[52]
6	Indonesia	10												[53]
7	Egypt	10												[67]
8	India	23												[91]
9	India	57												[58]
10	India	6												[74]
11	China	25												[59]
12	India	97												[66]
13	India	68												[49]
14	Saudi Arabia	7												[57]
15	Saudi Arabia	7												[48]
16	Pakistan	90												[73]
17	Nigeria	31												[72]
18	Indonesia	8												[54]
19	India	7												[55]
20	India, Laos, Philippines, Taiwan, Tanzania, Thailand, USA	18												[32]
21	India	97												[88]
22	India	36												[56]
23	India	34												[47]
24	India, Laos, Philippines, Taiwan, Tanzania, Thailand, USA	18												[33]
25	India	12												[45]
26	Ethiopia	5												[46]
27	India, Malaysia, Taiwan, Tanzania, Thailand, USA	20												[71]
28	India	8												[65]
29	India	12												[4]
30	Asia, Africa, North and South America, and the Caribbean	161												[87]
31	Brazil	16												[70]
32	Tanzania	78												[40]
33	Tanzania	87												[69]
34	India, Myanmar	24												[86]
35	India	77												[44]
36	India	28												[43]
37	India, Kenya, Malawi	140												[62]

The mapping with green represents the techniques used for the study.

#### 5.3.10. Comparative Efficacy of Different Molecular Markers

Several studies employed either two (RAPD and SSR [72], CytP450 and RAPD [55], ISSR and SRAP, and ISSR and RAPD [49]) or three (ISSR, ITS, and SCoT [67] and CytP450, ISSR, and RAPD [65]) different molecular markers for the comparative analysis of their potency in detecting polymorphism in MO accessions collected from different agroclimatic regions across the world. Together, these studies revealed the usefulness of morphological, nutritional, biochemical, and molecular markers for determining the genetic diversity in the accessions and advanced breeding lines of MO, which could be exploited for conservation, DNA fingerprinting, marker-assisted selection, cultivar development, and breeding program. A snapshot is presented providing an overview of the different markers employed for assaying the genetic diversity in MO accessions (Table 1). Although the desirable characteristics of molecular markers are their low cost for development and assay, even and frequent distribution throughout the genome, high reproducibility, moderate to highly polymorphic nature, and codominant inheritance, each of them is endowed with advantages albeit with some limitations [36] (Table 2).

## 6. Breeding and Yield

### 6.1. Breeding

MO varieties are broadly classified into perennial and annual (www.greenagrow.blogspot.com, accessed on 20 March 2021). Perennial types are generally propagated from cuttings but have several constraints (limited availability of suitable stem cuttings as planting material, long growing period before reaching maturity to produce pods, greater rainfall requirement, and, prone to pests and diseases) that limit their use for commercial production in areas with a shortage of water and short growing season (www.greenagrow.blogspot.com). Annuals, with a shorter life span, are seed-propagated, mature rapidly, adapt to varied climatic and soil conditions, have high yields, and exhibit significant variations in the fruit weight and the number of flowers/inflorescence and fruits/plant (www.greenagrow.blogspot.com). Annual and perennial MO are being extensively used for breeding with various desirable traits such as dwarf stature, suitable for leaf production, high-yielding types, high seed and oil content, and resistance to pests and diseases (www.agritech.tnau.ac.in). Tamil Nadu Agricultural University (TNAU) at Periyakulam, India has a collection of 85 accessions of MO and concerted breeding efforts have resulted in the development of promising high-yielding seed-grown annual varieties Periyakulam 1 (PKM-1) and Periyakulam 2 (PKM-2) (www.agritech.tnau.ac.in). PKM-1, developed through pure line selection, is an early variety of dwarf or medium stature, in the first year after planting reaches a height of 4 M, and produces flowers and pods six months after sowing. Therefore, PKM1 is the most widely planted variety for large-scale production of the drumsticks adapted to grow in the stubble of other crops harvested earlier with a yield potential of 50–54 tons/ha (www.agritech.tnau.ac.in). A full characterization of the oil produced from the seeds of PKM-1 showed high stability to the oxidative rancidity [92]. PKM-2 is suitable for intercropping with coconut and tropical fruit orchards requiring more water than PKM1 and produces ~240 fruits/tree with an average yield of 98 tons/ha (www.agritech.tnau.ac.in). Both PKM-1 and PKM-2 exhibit superiority over perennial types due to their adaptability to varied soil and climatic conditions and have replaced ~60% of perennial Moringa in the southern states of India that earlier dominated commercial production (www.agritech.tnau.ac.in). In India, several other varieties have been released for high production and quality of drumsticks (Anupama, Coimbatore 1, Coimbatore 2, G.K.V.K.-1, G.K.V.K.-2, and G.K.V.K.-3, and Rohit 1); a pest-resistant, adapted to grow in a hot dry region, and long-lived for 15–20 years (Bhagya [KDM-1]); a dwarf variety with a canopy of only 2.0–2.5 m in height, flowers early in 7–8 months, high yielding (250–300 pods/plant/year), and of good cooking quality (Dhanaraj [S 6/4]), and perennial varieties that can be maintained for up to 15 years without pruning (Moolanur), yield 1000–1200 pods per tree (Valayapatti), and with high germination rates and productivity (MS01 and MS02) (www.agritech.tnau.ac.in; https://sustainablebioresources.com, accessed on 20 March 2021). Chavakacheri and Chemmurungai are the ecotypes of the perennial Jaffna Moringa introduced from Sri Lanka into India (https://sustainablebioresources.com). Efforts are also underway at the University of Hawaii, USA to optimize the production of the leaf, pod, and oil (www.ctahr.hawaii.edu/RadovichT, accessed on 20 March 2021). Although China does not have a tradition of Moringa planting, Cuba does; therefore, their scientists have developed a cooperative research program at Xishuangbanna in China’s southwestern Yunnan province for developing improved varieties of MO (www.chinadaily.com.cn, accessed on 20 March 2021). At South China Agricultural University, Guandong, MO breeding program is focused on identifying functionally diverse genes associated with important agronomical traits [93]. Moringa Philippines Foundation (Philippines), AVDRC (Taiwan), Rural Development Initiative, and Moringa Community (Zambia) are other research centers across the world focused on the improvement of MO [3]. For the genetic improvement and development of superior cultivars, it is essential to assay the genetic diversity among the advanced breeding lines. Cytochrome P_450_, RAPD, and seed protein profile-based markers were efficacious in molecular fingerprinting and assessment of genetic variations among the advanced breeding lines of MO [55].

### 6.2. Yield

#### 6.2.1. Leaves

MO leaf production varies with the varieties, local agro-climatic conditions, and cultivations system ranging from intensive monocropping to intercropping, and from direct seeding to cuttings, irrigation, and fertilization (www.moringanews.org; accessed on 29 March 2022). Adequate fertilization and irrigation are pivotal for facilitating many cuttings per year (www.moringanews.org). High density planted MO in a well-drained fertile soil reaches a height of 1.5–2.0 m in 2–3 months and becomes suitable for harvesting the leaves manually with a sharp knife at 20–45 cm above the ground (www.moringanews.org). This method of harvesting promotes the development of new shoots, and leaves can be harvested subsequently every 35–40 days (www.moringanews.org). In Nicaragua (Central America), the average fresh leaf production was reported to be 174 metric tons (~30% of the total biomass harvested) from a plantation of 1 million Moringa plants/ha and nine cuttings per year over 4 years (www.moringanews.org). In Senegal (West Africa), fresh leaf production ranged from 45 metric tons/ha during the dry season and significantly increased to 115 metric tons/ha during the rainy season (www.moringanews.org). To avoid deterioration, freshly harvested MO leaves should not be heaped and are used as fodder for livestock, eaten as vegetables, and can also be frozen or canned (www.moringanews.org). About 8 kg of the fresh leaves (with stem removed) yields ~1 kg of dried leaf powder (www.moringanews.org). MO leaves, rich in various bioactive polyphenols, novel polysaccharides, phytochemicals, and secondary metabolites, are extensively used for their multiferous nutraceutical and pharmacological properties, as a functional food or other products for health care, and as an additive in animal diets to improve the meat quality [10,94,95,96,97,98,99]. Ultra-high-performance liquid chromatography with a diode array coupled to electrospray ionization mass spectrometry (UPLC-ESI-QTOFMS) analysis of the methanol extract from MO leaves revealed a significant influence of the seasonal variations on the production of secondary metabolites, and therefore autumn and summer were recommended to be the best harvesting seasons [100]. 

#### 6.2.2. Pods

Although MO pods ripen during the summer, sometimes the flowers and pod appear twice a year with two harvests during March–April, and July–September (www.agritech.tnau.ac.in). MO pods mature ~3 months after flowering and persist on the tree for several months (www.agritech.tnau.ac.in). The pod yield is normally low during the first 2 years but from the third year onwards, depending upon the variety, the number of pods produced/tree/year exhibits significant variation and ranges from 100 to 1600 (www.agritech.tnau.ac.in). Variety-specific variations are also reflected in the length (20–126 cm), weight (95–280 g) and yield (31 to 98 tons/ha/year) of the pod, and the number of seeds/tree/year ranges from 2000 to 3250 (www.agritech.tnau.ac.in). The seeds from the mature pods are dispersed by the wind, water, and/or seed-eating animals. In India, the commercial production of immature pods for processing is a large industry with ~1.2 million metric tons produced annually on 38,000 ha. The pods can be canned to preserve for later consumption. Gamma irradiation improved the shelf-life and physicochemical quality of the ready-to-cook pods [101]. The pods are fibrous and potent for treating digestive problems and their extracts/fractions exhibited antifungal activities against *Fusarium oxysporum* and *Rhizoctonia solani* [102]. However, the nutritive values of the green pods are often influenced by the elevation (1100 to 1700 m above sea level) during dry and rainy seasons [103].

#### 6.2.3. Oil

The oil content of MO seeds, which is regarded as an excellent natural cosmetic emollient [104], ranges from 35% to 45%, while that of de-hulled seed is ~42% (www.agritech.tnau.ac.in). The seed oil contains ~13% and ~82% saturated and unsaturated fatty acids, respectively (www.agritech.tnau.ac.in). The free fatty acid varies from 0.5% to 3% of the total oil content and has a particularly high level of oleic acid (~70%) followed by palmitic acid (~9.0%) [104,105] (www.agritech.tnau.ac.in). Since high oleic acid MO seed oil has a low melting point, lacks plasticity, and is not suitable for food products, its enzymatic interesterification with palm kernel oil and palm stearin is a viable option for yielding harder fat stocks with desirable physical and nutritional properties. The oil is conventionally extracted from the seeds by either mechanical pressing or solvent extraction at 40 °C with a specific mass in the range of 910–970 kg.m^3^ [106]. The extractive yield by the solvent (~35–41%) is relatively higher compared with the mechanical pressing (~26%). Moringa seed oil extracted by mechanical and/or solvent extraction generates diverse products suitable for both biodiesel and food industries [107]. MO seed oil remains stable for about six months of storage, after which it began to deteriorate [108]. From a health perspective, MO seed oil is rich in Δ5-avenalsterol, campesterol, phytosterols, β-sitosterol, stigmasterol, and α-tocopherol, and has a high resistance to oxidative rancidity [108].

## 7. In Vitro Propagation

MO has been extensively used for various applications worldwide, leading to a gradual decline in its biodiversity and natural population [109]. Although plant propagates easily by seeds and stem cuttings, the percent germination of the former is often low, and the plants grown from the latter show high mortality and low yield [110]. Moreover, the susceptibility of MO to several insect infestations and fungal infections leads to the foliar damage and loss of biomass exerting adverse influence on the overall yield and nutritional and commercial values. Therefore, in vitro propagation of MO provides a viable alternative for both conservation and clonal multiplication of genetically identical and infection-free elite germplasm year-round in a limited space and time under controlled growth conditions to produce plant tissues and/or the commercially important myriad of engineered bioactive compounds to meet the ever-growing global demand. The earlier reports on in vitro propagation of MO were in the 1990s followed by several other reports up to the early 2020s [111,112]. These studies were largely focused on optimizing the shooting and rooting efficacy of various explants by testing different combinations and concentrations of plant growth regulators (*PGRs*). The explants were taken either from the plant growing in the natural habitat or from its in vitro aseptically germinated seedlings. The nodal segments were used as explants in most of these studies, while a few of them used shoot apices, leaves, or immature seeds.

Murashige and Skoog (MS) has been the preferred basal medium [113] for most of the studies on in vitro propagation of MO with a notable exception of the use of Woody Plant Medium (WPM) in one of the studies [114]. WPM contains lesser amounts of ammonium and total nitrogen than MS [115]. Different combinations and concentrations of PGRs were used for the induction and multiplication of the shoot buds. For most of the studies, synthetic cytokinin 6-benzylaminopurine (BA) was used alone (0.5–1.0 mg/L) or in combination with naturally existing kinetin (Kn) (0.2–1.0 mg/L), naturally existing auxin indole-3-acetic acid (IAA) (0.2 mg/L), synthetic auxin naphthalene acetic acid (NAA) (0.05–1.0 mg/L), and gibberellic acid (GA3) (1.0 mg/L). Early shoot senescence was observed that restricted subsequent growth during in vitro culture [23]. Multiplication of shoots on the MS medium supplemented with BA resulted in shoot vitrification, which led to chlorosis, inhibited shoot formation, development of necrosis at the shoot tip, and formation of friable calli at the base of cultured explants [116]. However, a significant reduction in the shoot vitrification and its improved multiplication was observed when the medium was supplemented with AgNO_3_, which is a potent anti-ethylene agent [116]. The pivotal role of AgNO_3_ in the shoot multiplication was further corroborated [22]. Variations in the number of shoots per explant ranged from ~3 [114,117] to ~17 [22,118], which highlighted a significant influence of the variable growth conditions employed in these studies. Further, different concentrations and combinations of IAA, NAA, and indole-3-butyric acid (IBA) were used for inducing ~86–100% in vitro rooting of the regenerated shoots [114,117,118,119,120,121,122]. IBA was once thought to be a synthetic auxin, but later it was found to be an endogenous compound detected in taxonomically diverse plant species including Arabidopsis, maize, pea, potato, and tobacco suggesting its conserved role across the species [123].

**Figure 3:**
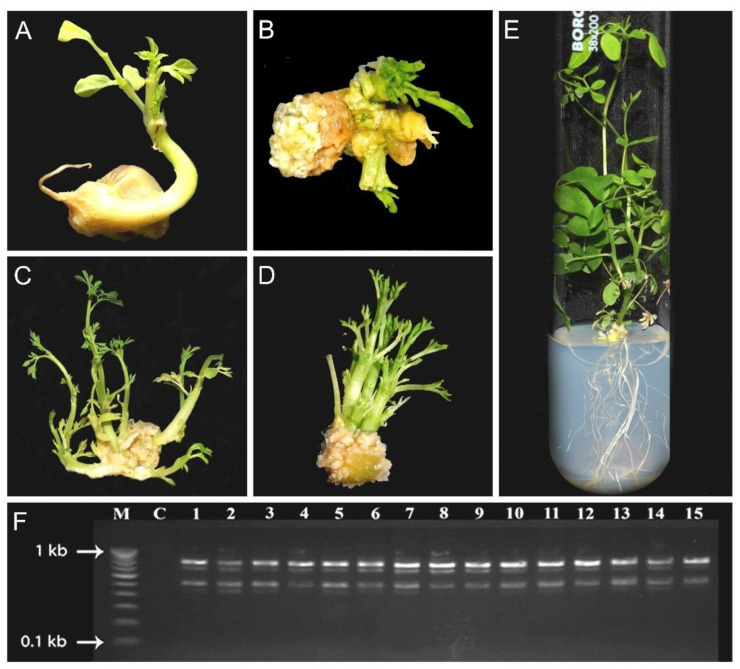
In vitro propagation of MO. (**A**,**B**) Multiple shoot formation from the decapitated seed (**A**) and nodal segment (**B**) cultured on MS medium with BA (0.5 mg/L). (**C**,**D**) Multiple elongated shoot formation from the nodal segment cultured on MS medium with (**C**) BA (0.5 mg/L) + Kn (1 mg/L) and (**D**) BA (1 mg/L) + Kn (0.5 mg/L). (**E**) The rooting response from in vitro regenerated shoots on one-half-strength MS medium with IAA (0.1 mg/L). (**F**) DNA amplification profile obtained with RAPD marker (OPT-1) for the clonal fidelity assessment of 1–15 regenerated plants (M = marker lane, C = control lane without DNA) [S.L. Kothari, unpublished work].

The regenerated rooted plantlets were placed gently in the pots and maintained under controlled conditions in the growth chamber for ~2 weeks, and then potted plantlets (covered with polythene bags) were transferred to the greenhouse (shaded) for a few weeks for acclimatization, which resulted in the successful survival of ~90–95% plantlets [22,114,118,119,120,121,124]. No abnormality in growth and morphology was observed in vitro-generated plantlets [119]. The genetic fidelity of the regenerated plantlets was ascertained by SSR, ISSR, RAPD, and Random amplified microsatellite polymorphism (RAMP) markers [22,116,118,124]. In vitro propagation of MO by using different explants (decapitated seed and nodal segment) and the effects of PGRs on shooting and rooting of the nodal segment cultured on MS medium were investigated (Figure 3A–E; S.L. Kothari, unpublished work). RAPD analysis revealed the clonal fidelity of the regenerated plants (Figure 3F) (S.L. Kothari, unpublished work). Compared with the wild-type plants, in vitro-generated plants revealed higher concentrations of α-tocopherol and total carotenoids indicative of their higher nutritional values [120], steroidal sapogenins (diosgenin and tigogenin) with antioxidant potential [121], precursor benzyl glucosinolate [125], and peroxidase enzyme involved in defense mechanism [117]. Together, these studies demonstrated the efficacy of in vitro propagation for generating disease-free nutritionally superior plants at a large scale for commercial purposes, maintaining the germplasm, and potentially augmenting the profiles of the bioactive constituents. A snapshot is presented providing an overview of different studies on in vitro propagation of MO (Table 3).

## 8. Trade

**Figure 4:**
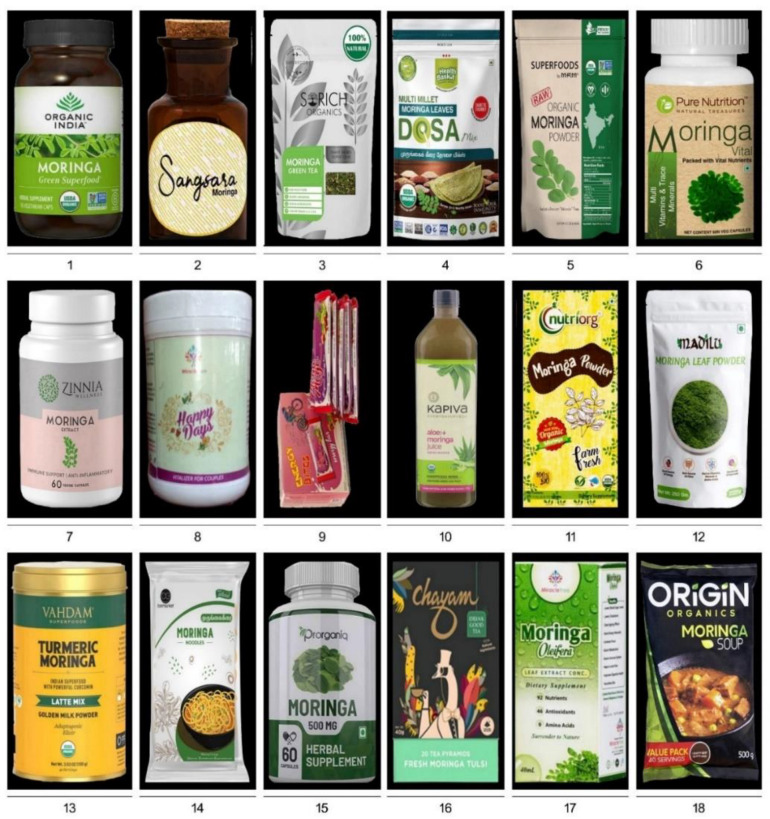
Moringa commercial functional food products. Details of products 1–18 are listed in Table 4.

**Figure 5:**
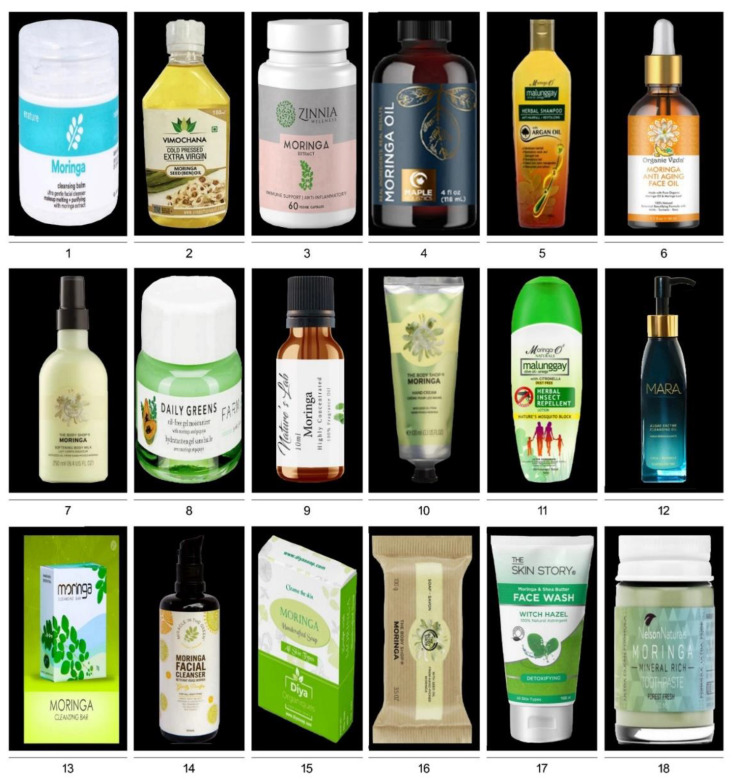
Moringa commercial non-food products. Details of products 1–18 are listed in Table 5.

MO offers a wide array of business opportunities to investors in producing various functional foods and non-food products [126,127,128,129]; (https://www.entrepreneurindia.co/blog-description/10225/manufacturing_of_moringa_oleifera_products; accessed on 2 April 2022) (Figure 4 and Figure 5; Table 4 and Table 5). MO’s various parts and/or products are exported across the globe [130]. India is the largest producer of MO, contributing 41% of the global production, followed by other tropical countries, i.e., Western Africa (33%), the Philippines (12%), China (8%), and Venezuela (6%) [131]. India meets ~80% demand for various MO products [131]. In 2017, India exported 11, 81,468 tonnes of various MO parts and/or products worth ~Rs 46.24 crores (~6.25 million US$) [131]. Among the Moringa products, the demand in the international market is very high for leaves, and India exported INR 11.61 crores (~1.56 million US$), INR 14.6 crores (~1.9 million US$), and INR 2.50 crores (~0.33 million US$) in 2014, 2015, and 2016 (January and February), respectively [132]. There are ~2135 Indian export ports (air, sea, an inland container depot [ICD], and a special economic zone [SEZ]) for Moringa products (https://www.seair.co.in/moringa-export-data.aspx; accessed on 2 April 2022). India is exporting Moringa products to various countries in Asia (Cambodia, China, Indonesia, Iraq, Israel, Japan, Kuwait, Lebanon, Malaysia, Mauritius, Pakistan, Philippines, Saudia Arabia, Singapore, South Korea, Sri Lanka, Taiwan, Thailand, United Arab Emirates, and Vietnam), Africa (Algeria, Botswana, Cameroon, Chad, Congo Democratic, Egypt, Ethiopia, Ghana, Guinea, Kenya, Namibia, Nigeria, Seychelles, South Africa, and Zambia), Australia/Oceania (Australia, New Zealand, Papua New Guinea, and the Solomon Islands), Europe (Austria, Belgium, Bulgaria, Croatia, Czech Republic, Finland, France, Germany, Hungary, Italy, Latvia, Lithuania, Netherlands, Poland, Portugal, Serbia, Slovakia, Slovenia, Spain, Sweeden, Switzerland, United Kingdom), transcontinental (located in Asia (97%) and Europe (3%)) (Turkey), North America (Bahamas, Canada, El Salvador, Haiti, Mexico, and the USA), and South America (Argentina, Brazil, Chile, Colombia, Paraguay, Peru, Uruguay, and Venezuela) [132] (https://www.seair.co.in/moringa-export-data.aspx; accessed on 2 April 2022) (Figure 1). Among the European countries, Germany has the largest market for Moringa products, followed by the United Kingdom, France, Netherlands, Italy, and Spain (www.cbi.eu/market-information/natural-ingredients-health-products/moringa/market-potential; accessed on 2 April 2022). In India, the leading manufacturers and exporters are Miracle Tree Life Science (MTLS) and Green India Future (GIF), offering a good quality value-added assortment of various Moringa products [130]. MTLS exports ~25% of their Moringa products to Malaysia, and *Wunder mix* (100 gm for ~700 INR [~10 US$]) exported to Germany has earned a good repute for the company [130] (www.indiamart.com/miracletreelifescience; accessed on 2 April 2022). Apart from MTLS and GIF, several other companies in India are also producing a wide range of Moringa products that aid in digestion, boost immunity, reduce acidity and inflammation, relax muscles, and with anti-aging, antioxidant, and skincare properties (Figure 4 and Figure 5; Table 4 and Table 5). In the Philippines, Moringaling Philippines Foundation, Inc. (MPFI) is a network organization which supplies Moringa products to consumers, exporters, farmers, and health enthusiasts (http://moringaling.global; accessed on 2 April 2022). African countries (Ghana, Kenya, Mozambique, South Africa, and Zambia) have also entered the global export market for various Moringa products (www.cbi.eu/market-information/natural-ingredients-health-products/moringa; accessed on 2 April 2022). In Zambia, Moringa is organically grown and supplied to both local and international markets by Moringa Initiative (https://moringainitiative.com; accessed on 2 April 2022). Although India enjoys great access to Moringa products among the global partners, some consignments have been rejected due to phytosanitary issues and/or not conducive to the stringent specifications and regulations laid out by the importing countries [130,132]. A fledgling Moringa industry is also faced with several uphill challenges by the small-holder farmers from developing *countries* like *Nigeria* and the Philippines in translating backyard production to global commercialization due to several constraints including seasonality in yield, poor sheds/storage facilities, and processing equipment at the production zone, lack of solar drying facility, inadequate financial resources, higher tax due to goods and services act, transportation problems, and lack of sufficient knowledge towards stringent quality control measures, regulatory approval, and international trade and market [130].

## 9. Health Concerns

Several studies have shown the efficacy of MO as an alternative source of nutrients and for the treatment of various diseases, albeit with a few apprehensions. Therefore, there is urgent need for contingent legal regulations, stringent risk assessment, consumption patterns, attitudes, and beliefs toward MO products [133,134,135,136]. For instance, the study conducted to examine the consumption behavior of MO leaves (MOL) and MO pods (MOP) of the adult population in Mauritius revealed some disagreement about its potency in managing hypertension [137]. In a clinical study, lowering of postprandial blood pressure was observed after the consumption of the cooked MOL [138]. Since MOL consumption can lower blood pressure, it is not advisable to mix it with medications for treating patients with high blood pressure (https://www.verywellhealth.com/the-uses-and-benefits-of-moringa-4149435; accessed on 20 March 2021). The consumption of MOL could also trigger hypoglycemia in diabetic patients [139,140]. Further, it is not prudent for patients to consume MOL when they are being treated with blood-thinning medication like warfarin (https://www.medindia.net/patients/lifestyleandwellness/side-effects-of-moringa.htm; accessed on 20 March 2021). The MOL has laxative properties and when consumed in large quantities can cause diarrhea, [140], gaseous distension, stomach upset, nausea, and activation of the pharyngeal reflex (https://www.medindia.net/patients/lifestyleandwellness/side-effects-of-moringa.htm; accessed on 20 March 2021). Levothyroxine is a medicine for treating thyroid hormone deficiency (https://www.drugs.com/levothyroxine.html). The MOL interferes with the absorption of levothyroxine and can aggravate hypothyroidism (https://www.webmd.com/vitamins/ai/ingredientmono-1242/moringa; accessed on 20 March 2021). MO roots (MOR) contain toxic alkaloid spirochin [141] and its oral consumption can cause nerve paralysis [142]. The chemicals in MOR, bark, and flowers trigger uterus contraction causing miscarriages, have anti-fertility properties, and are thus not recommended during pregnancy (https://www.medicalnewstoday.com/articles/319916; accessed on 20 March 2021). MOP may not be advisable for infants as it may cause infantile paralysis or convulsions [143]. Cells can be mutated by the chemicals isolated from the roasted seeds of MO (https://www.asbestos.com/blog/2019/11/26/moringa-tree-cancer-research; accessed on 20 March 2021). More detailed information about the side effects of MO on human health could also be found on these blogs:

- https://www.healthifyme.com/blog/moringa-health-benefits-side-effects-need-aware; accessed on 20 March 2021.
- https://www.medindia.net/patients/lifestyleandwellness/side-effects-of-moringa.htm; accessed on 20 March 2021.

Stephen Daniells reported on 20 June 2019 that the regulatory agency ANVISA (Agência Nacional de Vigilância Sanitária) in Brazil has prohibited the use of MO in food and supplements since there is no evaluation and proof of safety (https://www.nutraingredients-latam.com/Article/2019/06/20/ANVISA, accessed on 20 March 2021). However, many of the reported concerns on the side effects of different parts of MO are rather anecdotal and merit a pragmatic approach. A non-human living animal model, often genetically engineered, is used for deciphering various disease processes without causing any actual risk to human health. *Cavia porcellus* (guinea pigs), *Mus musculus* (mouse), and *Rattus norvegicus* (rats) mimic human diseases and are thus extensively used as model organisms for empirical evidence. Therefore, the use of model organisms is an attractive paradigm to have a better understanding of the various MO-mediated health hazards. Goitrogens are substances that interrupt the production of thyroid hormones by intervening with iodine (I_2_) uptake by the thyroid gland (https://en.wikipedia.org/wiki/Goitrogen, accessed on 20 March 2021). It was hypothesized that prolonged consumption of MOL (a rich source of goitrogens) could potentially cause hypothyroidism [144]. The authors fed the adult male albino rats for 1–2 months, which led to the development of hypothyroidism and thus validated their assumption. MOL extract had an abortive effect on rats when treated for 10 days after insemination [145]. Constant administration of the methanolic extract of MOL in healthy adult male Wistar rats could also damage the liver and kidney [146]. High-dose oral administration of the MOR aqueous extract to the rats resulted in antifertility [147]. The liver and kidney functions were also affected in mice administered with the methanolic extract of MOR [148]. Furthermore, guinea pigs subjected to intraperitoneal injections of methanolic MOR for three weeks exhibited histo-architectural distortions in the liver and kidney [149]. These studies highlighted a need for stringent toxicity evaluation and cautious consumption of different parts of MO by humans as food and for the treatment of various ailments.

## 10. Conclusions

**Figure 6:**
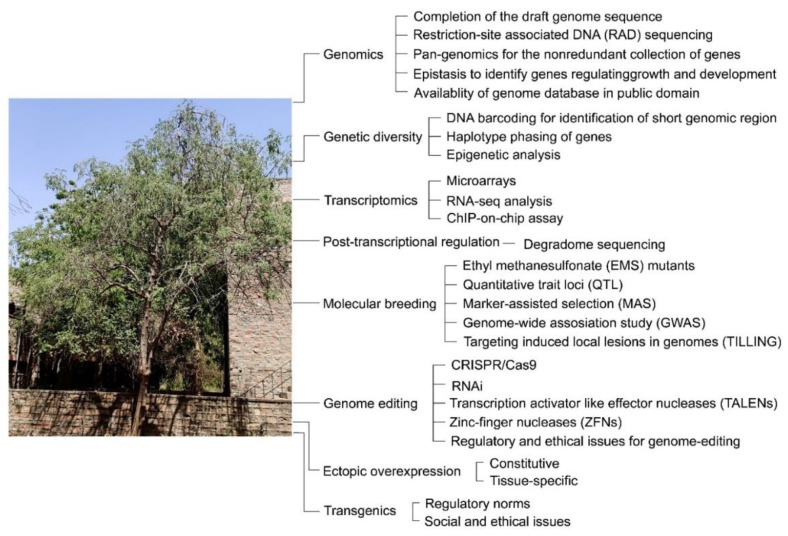
Schematic diagram showing perspective biotechnological interventions in MO for improvement of agronomic traits and conferring resistance to biotic and abiotic stresses.

Across the globe, a wide collection of MO germplasm resources is available with significant potential for their genetic improvement. Assessment of genetic diversity in MO accessions provides an opportunity for developing new and improved cultivars with desirable agronomic traits (high yield potential and resistance to various biotic and/or abiotic stresses). The current review was thus aimed at providing a landscape of various strategies that have been employed for assaying the genetic diversity in MO accessions collected from different agro-climatic regions of the world. Although several studies have used an array of morphological, biochemical, and phytochemical traits for determining the genetic diversity in MO accessions, these markers have several bottlenecks such as their limited numbers and are prone to be influenced by growth and development and various environmental factors. On the contrary, molecular markers are amenable to automation, provide wider genomic coverage, exhibit high reproducibility, and are independent of environmental perturbations. Therefore, several molecular markers with dominant and/or codominant inheritance (AFLP, ISSR, ITS, RAPD, SCAR, SCoT, and SRAP) and Cyt P450 were assayed for their potency in determining polymorphism in MO accessions. Among the molecular markers, RAPD was the most popular one and employed in ~42% of studies followed by SSR (~35%) and ISSR (~19%). However, one of the limitations of the dominant RAPD marker is its inability to differentiate the allelic differences of a gene in a heterozygous condition. SCAR marker circumvents the limitation of the RAPD marker by often converting it into a codominant and locus-specific marker. Several other molecular markers, e.g., allele-specific associated primer (ASAP), cleaved amplified polymorphic sequence (CAPS), directed amplification of minisatellite DNA (DAMD), diversity array technology (DArT), randomly amplified microsatellite polymorphisms (RAMPO), restriction landmark genomic scanning (RLGS), sequence tagged microsatellite (STM), single nucleotide polymorphisms (SNPs), and single-strand conformation polymorphism (SSCP) [36] could also be potentially useful for elucidating the genetic diversity among the MO accessions and thus merit detailed investigations. Identification of elite accessions of MO would provide a much-needed fillip towards generating them for desirable agronomic traits by conventional methods and/or molecular breeding complemented by significant advances in whole-genome assembly and a global transcriptomic analysis [150,151] (Figure 6). It is anticipated that superior MO accessions would have a commensurate influence on their repertoire of commercially viable functional food and non-food products for trade and commerce.

## Figures and Tables

**Table 2:** Characteristics of the molecular markers used for assaying the genetic diversity in MO accessions.

Molecular marker	Method of detection	Inheritance	Advantages	Limitations	References
AFLP	Hybridization-based	Dominant	Detect multiple loci High polymorphism High reproducibility	Large amount of genomic DNA required Labour-intensive Cost-prohibitive	[61]
ISSR	PCR-based	Dominant	High polymorphism Multiplexed banding profiles Can be converted into a sequence-tagged site (STS) marker	Low resolution on the agarose gel Non-homology of similar-sized fragments Low reproducibility	[63]
RAPD	PCR-based	Dominant	Low amount of DNA required and easy to assay High genomic abundance High polymorphism	False-positive Non-reproducibility Requires a high concentration of agarose gel for a better resolution	[68]
SCoT	PCR-based	Dominant	Targets the functional genes and their corresponding traits Possibility of finding new alleles among a given germplasm collection Highly polymorphic	Primer designing is constrained by the number of highly conserved nucleotides within the ATG region Primer length and annealing temperature do not ensure reproducibility	[75]
ITS	PCR-based	Codominant	Rapid and simple technique Useful for the assessment of phylogenies and ecological studies DNA barcoding at the generic or species level	The sum of the DNA fragment sizes generated from the restriction digestion could be larger than the original size of the ITS fragment Incomplete restriction digestion results in false-positive bands Paralogous gene copies	[79]
SCAR	PCR-based sequencing of the termini of the unique RAPD marker	Dominant and codominant	Locus-specific Highly reliable and reproducible Useful for genomic and cDNA fingerprinting and pyramiding of the genes	Requires sequence information for redesigning the PCR primers Laborious and time-consuming More SCAR markers are required for the discrimination of traits	[80]
SRAP	PCR amplification of ORFs	Dominant and codominant	Facile sequencing of the selected bands Simple, efficient, and effective for generating genome-wide fragments Coding sequences are targeted, which generates moderate numbers of codominant markers	Does not generate heterozygosity descriptors such as Hardy–Weinberg equilibrium. Diversity deciphered by SRAP markers does not reveal evolutionarily relevant systematic relationships Moderate throughput ratio	[84]
SSR	PCR-based	Codominant	Technically simple and high reproducibility High allelic diversity and relative abundance Detect adaptive variation-mediated functional diversity	High development costs and require polyacrylamide electrophoresis Presence of more null alleles and occurrence of homoplasy Not suitable to detect the variation within cultivars	[85]
Cyt P450	PCR-based	NA	Efficient marker for deciphering genetic diversity in plants comprising both genome-wide and functional regions Multi-allelic and locus-specific gene family High polymorphism	NA	[90]

NA: Not Available.

**Table 3:** Snapshot of different studies on in vitro propagation of MO.

S. No.	Explant source	Explant	Basal medium	PGR for shoot induction (mg/L)					No. of shoots/explant	PGR for root induction (mg/L)			References
				BA	Kn	IAA	NAA	GA3		IAA	NAA	IBA	
1	In vitro germinated seedlings	Nodal segments	MS	1	1				17.3			0.2	[118]
2	In vitro germinated seedlings	Nodal segments	MS	1					3.5			1	[117]
3	In vitro germinated seedlings	Nodal segments	WPM	1					3.2			0.49	[114]
		Shoot apices	WPM	1			0.5		12			0.25	
4	Plants growing in natural habitat	Nodal segments	MS	0.5					17.6		9.31		[22]
5	In vitro raised plantlets	Nodal segments	MS	0.2					8.3				[116]
6	In vitro germinated seedlings	Nodal segments	MS	1		0.2			1.9				[124]
7	In vitro germinated seedlings	Leaves	MS	0.8	0.2		0.05		4.4		0.1		[122]
8	Plants growing in natural habitat	Nodal segments	MS	1		0.2	1					3	[121]
9	Plants growing in natural habitat	Nodal segments	MS	0.5					4.4				[125]
10	In vitro germinated seedlings	Nodal segments	MS	1					9	0.5		1	[120]
11	In vitro germinated seedlings	Shoot apices	MS	0.5					4.6		0.05		[119]
12	Plants growing in natural habitat	Immature seeds	MS	1				1	4.7		0.5		[23]

6-benzylaminopurine = BA; Kinetin = Kn; Indole-3-acetic acid = IAA; Naphthalene acetic acid = NAA; Gibberellic acid = GA3; Indole-3-butyric acid = IBA. The mapping with green represents different PGRs used for the induction of shoot and root.

**Table 4:** Commercial details of Moringa functional food products.

S. No.	Product	Description	Ingredients	Company	Usage	Weblink
1	Moringa green superfood	Aids in detoxification and boosts energy	Moringa leaves and pullulan capsules	Organic India Private Limited, Barabanki, India	Four capsules a day or as directed by the physician	https://www.amazon.com/ORGANIC-INDIA-Moringa-Supplement-Capsules/dp/B00BGVLG64; accessed on 20 March 2021
2	Sangsara Moringa	Aids in detoxification and reduces inflammation	Moringa plant extract	Aadi Herbals (P) Ltd., Panchkula, India	Two capsules a day or as directed by the physician	https://www.lazada.com.ph/products/moringa-o2-herbal-anti-hairfall-shampoo-w-argan-oil-350ml-i790992964-s2406570028.html; accessed on 20 March 2021
3	Moringa green tea	Aids in detoxification and weight loss	Moringa leaves and green tea	Sorich Organics, Noida, India	As instructed on the pack	https://www.amazon.in/Sorich-Organics-Moringa-Green-100g/dp/B0742FGJMZ; accessed on 20 March 2021
4	Moringa leaf idly dosa chutney powder	Aids in digestion	Moringa leaves, asafoetida, Bengal gram, black gram, cumin, curry leaves, red chilli, and salt	Qualis foods, Erode, India	Spice mix for dosa and idly	https://www.thespiceclub.in/shop-by-products/gourmet-foods-grocerices/nutritious-chutney-powders/moringa-leaf-idly-dosa-chutney-powder-100g; accessed on 20 March 2021
5	Organic Moringa powder	Aids in digestion	Organic moringa leaves	MRM Nutrition, California, USA	NA	https://www.amazon.com/MRM-Organic-Moringa-Verified-Gluten-Free/dp/B015279T5W; accessed on 20 March 2021
6	Moringa vital	Aids in digestion and boosts immunity	Moringa leaf powder, calcium, gooseberry, multivitamins, pomegranate, spirulina, and wheat grass	Herbs Nutriproducts (P) Ltd., Mumbai, India	NA	https://purenutrition.in/moringa-vital-wholefood-multivitamins-trace-minerals.html; accessed on 20 March 2021
7	Moringa extract	Anti-ageing; reduces inflammation	Moringa leaves	Zinnia Wellness, Ahmedabad, India	Two capsules twice a day after meals	https://mednear.com/otc/zinnia-wellness-moringa-extract-veggie-capsule-6038c26f394900003d023703; accessed on 20 March 2021
8	Happy days	Boosts energy	Moringa flowers, leaves, seeds and velvet bean seed powder	Miracle Tree Life Science, Madurai, India	NA	https://www.indiamart.com/proddetail/moringa-happy-days-20470996533.html; accessed on 20 March 2021
9	Mogo organic energy bar	Boosts energy	Moringa leaves, alfalfa, almond, cardamom, dry ginger, ground nut, jaggery, and spirulina	Satvik Organics and Naturals, Bhavnagar	Snack any time of the day	https://www.indiamart.com/proddetail/mogo-organic-moringa-energy-bar-22972602188.html; accessed on 20 March 2021
10	Aloe + moringa juice	Boosts energy and reduces inflammation	Moringa freshly grown plant juice, aloe vera leaf juice, citric acid, and permitted class ii preservatives	Kapiva Ayurveda, Mumbai, India	Add 30 mL Aloe + moringa juice to glass of water	https://www.indianproducts.fr/product/kapiva-100-organic-aloe-vera-usda-moringa-juice-energy-booster-no-added-sugar-1-ltr; accessed on 20 March 2021
11	Moringa leaf powder	Boosts energy and vitamin nutrition	Moringa leaves	Madilu Organics, Bangalore, India	Add to beverage and food	https://www.amazon.in/Madilu-Moringa-Leaf-Powder-200grams/dp/B08QM6Q7GT; accessed on 20 March 2021
12	Moringa leaf powder	Boosts energy and vitamin nutrition	Moringa leaves	Madilu Organics, Bangalore, India	Add to beverage and food	https://www.amazon.in/Madilu-Moringa-Leaf-Powder-200grams/dp/B08QM6Q7GT; accessed on 20 March 2021
13	Turmeric Moringa	Boosts immunity	Moringa leaves, black pepper, stevia, and turmeric	VAHDAM Teas (P) Ltd., New Delhi, India	Mix with milk	https://www.vahdamteas.com/products/turmeric-moringa-latte-mix?variant=16523126997035.html; accessed on 20 March 2021
14	Moringa noodles	Boosts immunity	Moringa leaves and noodles	BE Market, Chennai, India	NA	https://www.bemarket.store/product/moringa-noodles-muringaikeerai-175gms; accessed on 20 March 2021
15	Moringa herbal supplement	Boosts immunity and reduces inflammation	Moringa leaves	Prorganiq, Chennai, India	NA	https://prorganiq.com/products/moringa.html; accessed on 20 March 2021
16	Fresh Moringa Tulsi	Enriched with vitamins and minerals	Moringa leaves, ginger, green tea, licorice, and tulsi	Darshananand Holdings (P) Ltd., Kolkata, India	Brew in warm or iced water	https://www.herbkart.com/product/chayam-moringa-tulsi-green-tea-with-ginger-20-pyramid-tea-bags; accessed on 20 March 2021
17	Moringa oleifera leaf extract conc.	Food supplement	Moringa leaves extract, shea butter, and witch hazel	Miracle Tree Life Science, Madurai, India	NA	https://www.indiamart.com/proddetail/moringa-energy-drops-15474872297.html; accessed on 20 March 2021
18	Origin organics Moringa soup	Health supplement	Moringa leaves and beef	Origin Organics, Indore, India	Soup mix	https://www.takealot.com/moringa-soup-hearty-beef-500g-value-pack/PLID69449837; accessed on 20 March 2021

NA—Information not available.

**Table 5:** Commercial details of Moringa non-food products.

S. No.	Product	Description	Ingredients	Company	Usage	Weblink
1	Moringa cleansing balm	Anti-aging and facial cleanser	Moringa seed extract, birch juice, safflower seed oil, sodium ascorbyl phosphate, and tocopheryl acetate	E Nature, Seoul, Korea	Massage the balm on the face in circular motions and rinse off with lukewarm water	https://www.haruharubeauty.com/us/enature-moringa-cleansing-balm.html; accessed on 20 March 2021
2	Moringa seed (Ben) oil	Anti-aging and health supplement	Moringa seed oil	Vimochana Oil, Madurai, India	NA	https://www.bigbasket.com/pd/40126915/vimochana-oil-moringa-seed-cold-pressed-180-ml; accessed on 20 March 2021
3	Moringa extract	Anti-aging; reduces inflammation	Moringa leaves	Zinnia Wellness, Ahmedabad, India	Consume two capsules twice a day after meals	https://mednear.com/otc/zinnia-wellness-moringa-extract-veggie-capsule-6038c26f394900003d023703; accessed on 20 March 2021
4	Moringa oil	Anti-aging and skin healing properties	Moringa plant extract	Maple Holistics, NJ, USA	NA	https://www.u-buy.com.tw/en/product/37RD2HM-moringa-oil-for-hair-skin-and-nails-highly-absorbent-moringa-oleifera-hair-oil; accessed on 20 March 2021
5	Moringa herbal shampoo	Anti-hair loss shampoo	Moringa plant, argan oil, calcium, vitamins A, B complex, and E	Moringa-O2, Metro Manila, Philippines	Apply on wet hair, gently massage on scalp, and rinse	https://www.lazada.com.ph/products/moringa-o2-herbal-anti-hairfall-shampoo-w-argan-oil-350ml-i790992964-s2406570028.html; accessed on 20 March 2021
6	Moringa anti-aging face oil	Anti-wrinkle face oil	Moringa leaves and seeds, amla fruit, cassia auriculata, rose petal, sesame oil, and turmeric	Organic Veda, Virginia, USA	Apply oil on face and massage gently	https://www.amazon.com/Organic-Veda-Moringa-Anti-Aging/dp/B07HWSL2QW; accessed on 20 March 2021
7	Moringa softening body milk	Body lotion	Moringa seeds and benne oil	The body shop, Sussex, UK	Apply Moringa body milk after the shower all over your body	https://www.thebodyshop.in/moringa-milk-body-lotions.html; accessed on 20 March 2021
8	Daily greens	Face moisturizer	Moringa seed extract, hyaluronic acid, papaya extract, and polyglutamic acid	Farmacy Beauty, New York, USA	Gently apply to a clean face	https://www.sephora.com/product/farmacy-daily-greens-oil-free-gel-moisturizer-with-moringa-papaya-P458209; accessed on 20 March 2021
9	Moringa fragrance oil	Fragrance oil	Moringa leaves, bergamot, clary sage, geranium, green peppers, sandalwood, and wild spearmint	Natural Sisters, New York, USA	NA	https://naturalsistersproducts.com/products/moringa-fragrance-oil-fragrance-oil-moringa-33oz-1oz; accessed on 20 March 2021
10	Moringa hand cream	Hand cream	Moringa flower infusion and seed oil	The Body Shop International Ltd., Sussex, UK	NA	https://montenegro.desertcart.com/products/64627209-the-body-shop-moringa-hand-cream-100ml; accessed on 20 March 2021
11	Herbal insect repellent	Mosquito repellent	Moringa plant, aloe vera extract, oils from andiroba seed, citronella, eucalyptus, lemon, lemongrass	Moringa-O2, Metro Manila, Philippines	Apply lotion on hands and legs	https://shopee.ph/Moringa-O2-Herbal-Insect-Repellent-Lotion-(Deet-Free)-50ml-i.264554677.6135482445; accessed on 20 March 2021
12	Algae enzyme cleansing oil	Skin cleanser	Moringa seed oil, aloe vera extract, grapefruit extract, jojoba seed oil, papaya fruit extract, and sunflower seed oil	MARA beauty, California, USA	Apply two pumps on hand, massage into skin for 20–30 s, and rinse clean	https://www.amazon.com/MARA-Natural-Cleansing-Non-Toxic-Plant-Based/dp/B08NHVYP3L; accessed on 20 March 2021
13	Moringa cleansing bar	Skin cleanser	Moringa leaves	Frigoscan Post Harvest Technologies (P) Ltd., Chennai, India	NA	https://www.thiruveer.com/product/moringa-soap; accessed on 20 March 2021
14	Moringa facial cleanser	Skin cleanser	Moringa seed oil, aloe vera, grapefruit, and honey	Miracle in the green, Florida, USA	NA	https://www.amazon.com/Miracle-Green-Moringa-Facial-Cleanser/dp/B07VMVBK9T; accessed on 20 March 2021
15	Moringa handcrafted soap	Skin cleanser	Moringa leaves, coconut oil, neem oil, nilgiri oil, and shea butter	Diya Organiques, Kanchipuram, India	NA	https://diyasoap.com/product/moringa-soap; accessed on 20 March 2021
16	Moringa soap	Skin cleanser	Moringa seed oil, glycerine, palm oil, and soybean oil	The Body Shop International Ltd., Sussex, UK	NA	https://clicks.co.za/the-body-shop_moringa-soap/p/86978; accessed on 20 March 2021
17	Moringa & Shea butter face wash	Skin cleanser	Moringa leaves, shea butter, and witch hazel	Rahul Enterprises, Thane, India	Dampen face and neck and apply following by gently massaging, and then wash off	https://www.theskinstory.in/the-skin-story-moringa-shea-butter-facewash-detoxifier-100-ml.html; accessed on 20 March 2021
18	Moringa mineral-rich toothpaste	Toothpaste	Moringa leaves and seed oil, bentonite clay, colloidal silver, iodine, sea salt, and spirulina	Nelson Naturals, British Columbia, Canada	NA	https://www.carbonboutique.com/moringa-mineral-rich-toothpaste-60ml.html; accessed on 20 March 2021

## Data Availability

The study did not report any data.
